# Significant role of the choroidal outer layer during recovery from choroidal thickening in Vogt-Koyanagi-Harada disease patients treated with systemic corticosteroids

**DOI:** 10.1186/s12886-015-0171-3

**Published:** 2015-12-18

**Authors:** Kiriko Hirooka, Wataru Saito, Kenichi Namba, Kazuomi Mizuuchi, Daiju Iwata, Yuki Hashimoto, Susumu Ishida

**Affiliations:** Department of Ophthalmology, Hokkaido University Graduate School of Medicine, Sapporo, Japan; Department of Ocular Circulation and Metabolism, Hokkaido University Graduate School of Medicine, Nishi 7, Kita 15, Kita-ku, Sapporo, 060-8638 Japan

**Keywords:** Choroidal inner layer, Choroidal outer layer, Choroidal thickness, Vogt–Koyanagi–Harada disease

## Abstract

**Background:**

Which of the choroidal layers suffers the most extensive morphological changes during the course of Vogt–Koyanagi–Harada (VKH) disease is still unknown. The aim of this study was to investigate the relationship between total thickness and the thickness of inner or outer layers in the choroid during systemic corticosteroid therapy in patients with VKH disease.

**Methods:**

This retrospective case series included 15 eyes of 10 patients with treatment-naïve VKH disease (4 men and 6 women; mean age, 41.4 ± 14.7 years) received systemic corticosteroid therapy. Whole, inner, and outer choroidal thickness was measured manually at 1 week and at 1 and 3 months after initiation of systemic corticosteroid therapy using enhanced depth imaging optical coherence tomography. The mean thickness values of the layers were compared at each stage.

**Results:**

Compared with the 1-week baseline, the mean whole and outer choroidal layer thicknesses were significantly lower at 1 (*P* = 0.008 and 0.03, respectively) and 3 months (*P* = 0.008 and 0.02, respectively), whereas the inner layer did not significantly thin. Importantly, there was a significant positive correlation between the rates of change of whole and outer layer thickness from 1 week to 3 months (*R* = 0.9312, *P* < 0.0001), but not between the rates of whole and inner layer thickness changes.

**Conclusions:**

The thinning of total choroidal thickness observed after treatment with corticosteroids strongly correlated with outer layer thinning, suggesting that the choroidal outer layer is the primary target in acute-stage VKH disease.

## Background

Vogt–Koyanagi–Harada (VKH) disease is a multisystemic disease that affects systemic tissues containing melanin [1]. In the eye, this disease is characterized by panuveitis with serous retinal detachment (SRD) and an underlying circulation disorder in the choroid [2, 3], a tissue known as the primary target of the disease. Choroidal thickening at its acute uveitic stage subsides after systemic corticosteroid therapy [4] but often reappears along with recurrences of SRD [5] or even only anterior-chamber inflammation [6], suggesting that choroidal thickness could be a useful surrogate marker to quantitatively evaluate the disease activity.

Histopathologic findings in VKH disease are characterized by diffuse infiltration of lymphocytes with focal granulomas in the choroidal stroma [7], suggesting that the choroidal stroma is primarily affected in the acute stage of this disease. Using laser speckle flowgraphy (LSFG), we found that choroidal blood flow velocity in tissues deeper than the choriocapillaris decreases in the acute stage of VKH disease [3, 8]. The observation may indicate functional damage of the choroidal stroma in this disease. To our knowledge, however, there are no reports of which layers of the choroid are mainly affected morphologically in acute VKH disease.

Recent results on choroidal layer-by-layer measurements with spectral-domain optical coherence tomography (OCT) in healthy eyes demonstrated mean thickness values of 52.9, 204.3, and 256.8 μm for the inner (choriocapillaris to medium vessels), outer (choroidal large vessels), and whole layers, respectively [9]. Subsequently, several studies have reported separately measured choroidal layer thickness values in eyes with geographic atrophy, tilted disc syndrome, and Stargardt disease [10–13]. However, there are no studies that examined the thickness of choroidal inner or outer layers in patients with acute VKH disease. We herein report the inner and outer choroidal layer thickness changes after systemic corticosteroid therapy for VKH disease.

## Methods

### Patients and diagnosis

This retrospective case-series study included 15 eyes from 10 patients with treatment-naïve VKH disease (4 men and 6 women; mean age, 41.4 ± 14.7 years) who visited the uveitis clinic of Hokkaido University Hospital from May 2013 to November 2014. Patients’ choroidal layer-by-layer thickness changes were examined by enhanced depth imaging (EDI)-OCT (RS-3000 Advance; NIDEK, Gamagori, Japan) for up to 3 months after the start of systemic corticosteroids. VKH disease was diagnosed according to the criteria of Sugiura [14] and the VKH Disease Committee [15]. Exclusion criteria were presence of sunset-glow fundus, bullous retinal detachment, or myopia severity > −6 D. Eyes with OCT images that were not scanned at the same site in each stage were also excluded from this study, although all patients included had bilateral ocular involvement. None of the patients had any medical or ocular history, including systemic or ocular hypertension and ocular trauma or surgery. EDI-OCT images used in this study include those published earlier [8]. The current study was approved by the ethics committee of Hokkaido University Hospital (015–0162) and followed the tenets of the Declaration of Helsinki. Informed consent was obtained from subjects (Case 1 ~ 9) and the patient’s parents in Case 10 for chart review after the nature and possible consequences of the study had been explained.

### Treatment

Included patients received two regimens of high-dose systemic corticosteroid therapy according to the period of the patients’ first visit. In cases 1–4, administration of prednisolone was started at 200 mg/day. The tapering schedule for prednisolone has been described previously [8]. In cases 5–10, intravenous administration of methylprednisolone 1000 mg/day was initially initiated for three consecutive days. Then, oral prednisolone was tapered by schedule as follows: 10 days at 40 mg/day, 10 days at 30 mg/day, 10 days at 25 mg/day, 1 month at 20 mg/day, 1 month at 15 mg/day, 1 month at 10 mg/day, 2 months at 5 mg/day, and 1 month at 5 mg/every other day. In 4 eyes of 2 patients (Cases 9 and 10), the dose of prednisolone was temporarily increased, because recurrence of anterior chamber inflammation was noted within 3 months after the initiation of treatment. Oral cyclosporine was simultaneously administered for one patient with recurrence (Case 10).

### Ophthalmologic examination

On the initial visit, all patients underwent a complete ophthalmic examination, including decimal best-corrected visual acuity (BCVA), indirect ophthalmoscopy, fundus photograph, fluorescein angiography, indocyanine green angiography, spectral-domain OCT (cross-sectional retinal B-scans of 5 × 5 lines), and EDI-OCT. During follow-up, BCVA assessment and EDI-OCT were performed at pre-treatment, every week for 1 month after treatment, and every month thereafter.

### EDI-OCT

**Fig. 1:**
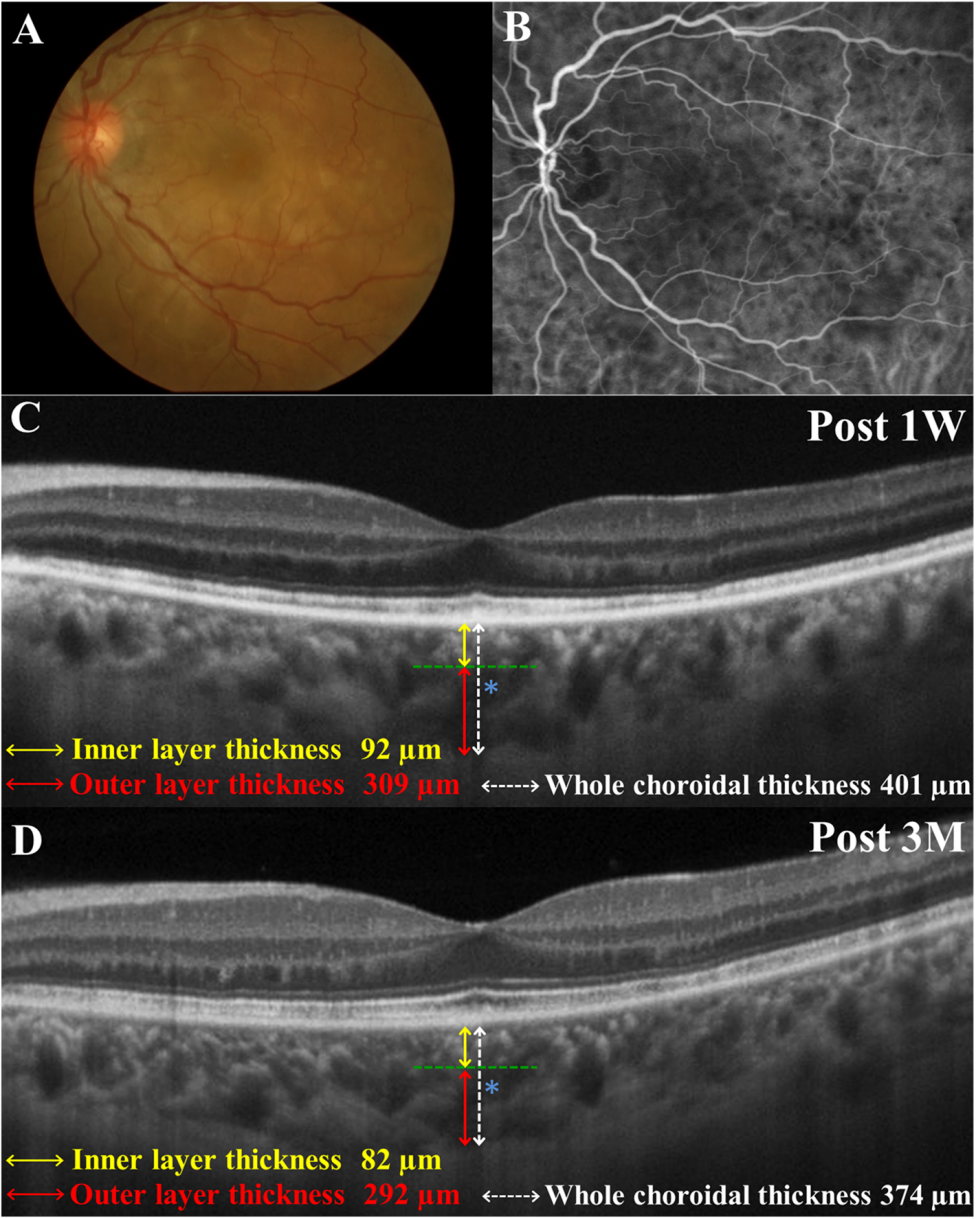
Images of the left eye in a patient (Case 3) with Vogt–Koyanagi–Harada (VKH) disease. **a** Fundus appearance at the initial visit showing serous retinal detachment involving the posterior pole and optic disc swelling. **b** Indocyanine green angiography at 5 min after the dye injection showing multiple hypofluorescent spots scattering beyond the posterior pole. **c**, **d** Horizontal images through the fovea on enhanced depth imaging optical coherence tomography (EDI-OCT) during systemic corticosteroid therapy. Choroidal outer layer thickness (red line) was measured from the inner border of the choroid-scleral junction to the innermost point (green line) of a large choroidal vessel (blue asterisk) observed in the closest proximity at the subfovea. Choroidal inner layer thickness (yellow line) was obtained via subtraction of the outer layer (red line) from whole thickness (white line). EDI-OCT at 1 week after treatment showing complete resolution of serous retinal detachment at the macular area, with subfoveal thickness values of 92, 309, and 401 μm for the inner, outer, and whole layers, respectively (**c**). Three months after the start of treatment, these thickness values decreased to 82, 292, and 374 μm for the inner, outer, and whole layers, respectively (**d**)

Choroidal thickness data on the inner, outer, and whole layers were manually collected using an EDI-OCT horizontal scan through the fovea, as described previously [9], at 1 week and 1 and 3 months after initiation of treatment. In brief, choroidal outer layer thickness (Fig. 1c, d, red line) was measured from the inner border of the choroid–scleral junction to the innermost point (Fig. 1c, d, green line) of a large choroidal vessel (Fig. 1c, d, blue asterisk) observed in the closest proximity at the subfovea [9]. Choroidal inner layer thickness (Fig. 1c, d, yellow line) was obtained via subtraction of the outer layer (Fig. 1c, d, red line) from whole thickness (Fig. 1c, d, white line) [9]. KH and YH evaluated OCT images in a masked fashion, not knowing any subject’s clinical information. Pre-treatment EDI-OCT data were eliminated from the analysis in this study, because the inner scleral border and choroidal vessel contours were hardly visualized due to severe choroidal swelling. We determined the statistical significance of the differences in average thickness values of each layer between each stage.

### Statistical analyses

The best-corrected visual acuity (BCVA) was converted to the logarithm of minimal angle of resolution (logMAR). The Friedman test and/or Wilcoxon’s signed-rank test were used to compare changes in logMAR BCVA and choroidal thickness in whole, inner, and outer layers. Spearman’s rank correlation test was used to determine the relationship between the rate of change in thickness for the whole layer and those for the inner or outer layers. For all tests, *P* < 0.05 was considered statistically significant.

## Results

### Ocular findings and retinal morphology

Ocular findings in eyes with VKH disease are summarized in Table 1. Before treatment, SRD was present in 10 of 15 eyes in the macular area, in the surroundings of the optic disc, or both. SRD was detected by OCT retinal scan in nine eyes especially in the macular area. SRD was undetectable in all eyes involved within 1 month after initiation of systemic corticosteroid therapy (Fig. 1c, d). The mean logMAR BCVA improved gradually after initiation of systemic corticosteroids and the value at 3 months was significantly improved compared with the 1-week baseline value (Table 2, *P* = 0.03, Wilcoxon’s signed-rank test).

**Table 1:** Clinical characteristics of Vogt-Koyanagi-Harada Disease Patients

							ICGA		FA	
No.	Age	Sex	R/L	Cell/Flare	SRD	Optic disc hyperemia	Fuzzy choroidal vessels	Hypofluorescent dark dots	Pinpoints Hyperfluorescence	Pleocytosis
1	34	F	L	+	−	−	+	+	−	+
2	56	F	R	−	+	+	+	+	+	+
			L	−	+	+	+	+	+	
3	31	F	R	−	+	+	+	+	+	+
			L	+	+	+	+	+	+	
4	43	F	R	+	+	+	+	+	+	+
5	57	F	R	+	+	+	+	+	+	+
			L	+	−	+	+	+	−	
6	24	M	R	−	+	+	+	+	+	+
7	44	M	R	−	−	+	+	+	−	+
8	46	F	R	+	−	+	+	+	−	−
9	64	M	R	+	+	+	+	+	+	+
			L	+	+	+	+	+	+	
10	15	M	R	+	+	+	+	+	+	+
			L	+	−	+	unknown	+	−	

*SRD* serous retinal detachment, *ICGA* Indocyanine green angiography, *FA* fluorescein angiography

**Table 2:** Changes in Choroidal Thickness

		logMAR BCVA				Whole layer thickness			Inner layer thickness			Outer layer thickness		
No.	R/L	Pre	1 W	1 M	3 M	1 W	1 M	3 M	1 W	1 M	3 M	1 W	1 M	3 M
1	L	−0.08	−0.08	not done	−0.18	753.5	379.0	422.0	158.0	134.0	119.5	595.5	245.0	302.5
2	R	−0.18	0.00	−0.18	−0.08	349.0	472.0	377.5	125.5	138.0	142.0	223.5	334.0	235.5
	L	−0.08	−0.08	−0.18	0.00	408.0	348.0	404.5	66.0	99.0	66.0	342.0	249.0	338.5
3	R	−0.18	0.00	0.00	−0.08	392.0	340.0	379.0	119.5	138.0	123.0	272.5	202.0	256.0
	L	0.70	0.05	0.05	−0.18	401.0	420.5	373.5	92.0	92.5	82.0	309.0	328.0	291.5
4	R	0.00	0.00	−0.18	−0.18	656.0	487.0	354.0	109.0	160.5	92.0	547.0	326.5	262.0
5	R	0.00	−0.08	0.00	−0.08	425.0	375.0	375.0	101.0	61.5	59.0	324.0	313.5	316.0
	L	−0.08	−0.18	−0.08	−0.08	470.0	441.0	478.0	78.0	90.5	80.0	392.0	350.5	398.0
6	R	0.10	0.00	0.30	−0.18	292.5	282.0	294.5	148.0	78.0	82.0	144.5	204.0	212.5
7	R	−0.08	0.70	0.05	−0.18	220.5	183.0	206.0	51.0	57.0	53.0	169.5	126.0	153.0
8	R	0.00	−0.08	−0.08	−0.08	342.5	144.1	317.0	131.5	127.5	138.0	211.0	16.6	179.0
9	R	0.22	0.00	0.00	−0.08	295.0	262.0	253.5	63.5	51.0	57.5	231.5	211.0	196.0
	L	1.30	0.22	0.00	−0.08	235.0	232.5	222.0	66.0	59.0	57.0	169.0	173.5	165.0
10	R	0.15	0.00	−0.18	−0.18	800.0	334.0	602.0	235.0	210.0	183.5	565.0	124.0	418.5
	L	−0.30	−0.30	−0.18	−0.18	800.0	262.0	307.0	143.5	134.0	127.5	656.5	128.0	179.5
	Average	0.10	0.01	−0.05	−0.12	456.0	330.8	357.7	112.5	108.7	97.5	343.5	222.1	260.2
	SD	0.39	0.21	0.13	0.06	192.7	99.1	97.4	46.3	43.7	37.9	164.5	94.5	80.3

*BCVA* best-corrected visual acuity

### Choroidal thickness

**Fig. 2:**
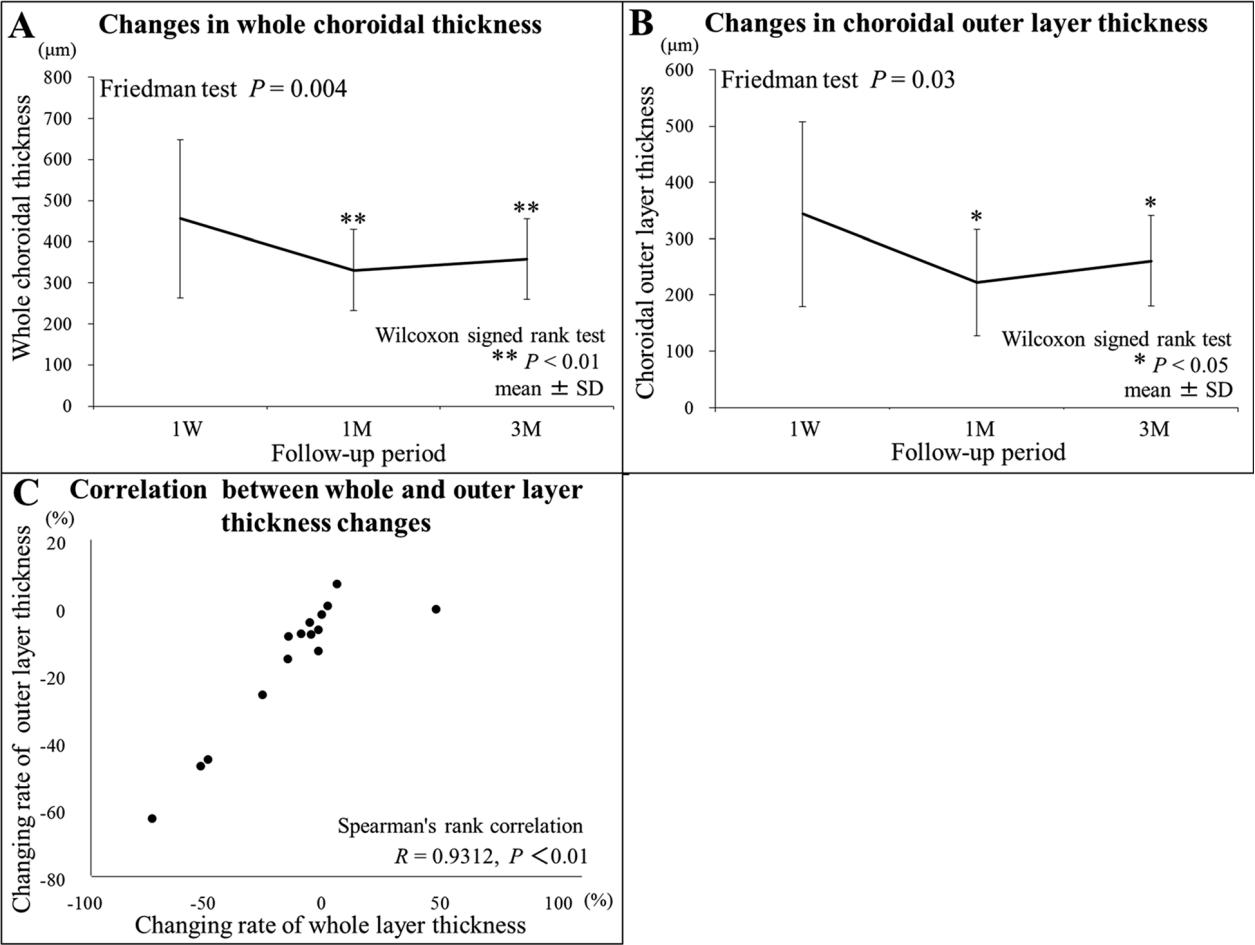
Choroidal layer-by-layer thickness changes during the 3-month period after the initiation of systemic corticosteroid therapy for Vogt–Koyanagi–Harada (VKH) disease. **a**, **b** Post-treatment choroidal thickness changes in the whole and outer layers measured with EDI-OCT. The mean whole (**a**) and outer (**b**) layer thickness significantly decreased at 1 and 3 months compared with the 1-week baseline. **c** Correlation between the whole and outer layers in the rate of change of thickness from 1-week baseline to 3 months. There was a significant positive correlation between the rates for the two layers

Thickness changes in the choroidal inner, outer, and whole layers are shown in Table 2. The mean thicknesses in the whole and outer layers decreased after initiation of systemic corticosteroid therapy. There was a statistically significant difference in thickness values of the whole and outer layers (Fig. 2a and b), but not in inner layer thickness during the 3 months follow-up period (Friedman test, *P* = 0.004, 0.03, and 0.22, respectively). Compared with the 1-week baseline, the mean whole (Fig. 2a) and outer (Fig. 2b) layers were significantly thinner at 1 (Wilcoxon signed rank test, *P* = 0.008 and 0.03, respectively) and 3 months (*P* = 0.008 and 0.02, respectively). As for the rate of change of thickness from baseline to 3 months, there was a significant positive correlation between the rates for the whole and outer layers (Fig. 2c; *R* = 0.9312, *P* < 0.0001), but not between the rates for the whole and inner layers (*R* = 0.4357, *P* = 0.1045).

## Discussion

In the present study, the choroidal outer layer significantly thinned concurrently with a strong correlation with the whole layer thinning during systemic corticosteroid therapy for VKH disease. In contrast, the inner layer did not change until the 3-month post-treatment visit and failed to show a correlation with the whole layer change.

In this study, we failed to measure the choroidal layer thicknesses before treatment, because the choroid was so thickened that thickness could not be measured accurately. At 1 week after treatment, however, the thicknesses of the whole, inner, and outer layers were higher than those reported for healthy eyes [9]. From these results, it is speculated that both the inner and the outer layers markedly thickened in the acute stage of VKH disease. This might be attributed to fluid deposition in both the layers, due to choroidal circulation impairment caused by choroiditis.

In the present study, the changes in whole layer thickness correlated with changes in the outer but not in the inner layer. Histopathologic observations revealed extensive infiltration of lymphocytes in the swollen choroidal stroma, but not in the choriocapillaris, in the acute stage of VKH disease [7], suggesting that its primary focus would be choroidal layers with relatively larger vessels. Our recent functional analysis with LSFG demonstrated that blood flow velocity in the choroidal stroma increases significantly after corticosteroid therapy, along with a significant correlation with the thinning of the choroidal whole layer in eyes with acute VKH disease [8]. Thus, the results of the present study and the previous observations suggest that it is the choroidal outer layer that contributes mainly to recovery from choroidal thickening in VKH disease treated with systemic corticosteroids. In other words, our current data may provide morphological evidence that the choroidal outer layer is primarily targeted in the acute stage of VKH disease, while the inner layer is secondarily involved.

The present study has some limitations. This study is retrospective and analyzed a small number of cases. In this study, the choroidal thickness was manually measured using OCT B-scan. In order to reduce measurement bias, further studies are needed to automatically measure the thicknesses of the choroidal inner and outer layers separately by development of swept source OCT C-scan.

## Conclusions

Our choroidal layer-by-layer measurements with EDI-OCT for VKH disease reveal that choroidal thinning as a result of systemic corticosteroid therapy is attributed to change in outer layer thickness. These results may provide morphological evidence that the choroidal outer layer is primarily affected in the acute stage of VKH disease.

## Abbreviations

BCVA: best-corrected visual acuity
EDI-OCT: enhanced depth imaging-OCT
logMAR: the logarithm of minimal angle of resolution
LSFG: laser speckle flowgraphy
OCT: optical coherence tomography
SRD: serous retinal detachment
VKH disease: Vogt–Koyanagi–Harada disease
